# Visfatin upregulates VEGF-C expression and lymphangiogenesis in esophageal cancer by activating MEK1/2-ERK and NF-κB signaling

**DOI:** 10.18632/aging.204762

**Published:** 2023-06-07

**Authors:** Chang-Lun Huang, David Achudhan, Po-I Liu, Yen-You Lin, Shan-Chi Liu, Jeng-Hung Guo, Chun-Lin Liu, Chih-Ying Wu, Shih-Wei Wang, Chih-Hsin Tang

**Affiliations:** 1 Graduate Institute of Biomedical Science, College of Medicine, China Medical University, Taichung 40402, Taiwan; 2 Department of Surgery, Division of Thoracic Surgery, Changhua Christian Hospital, Changhua 500, Taiwan; 3 Department of General Thoracic Surgery, Asia University Hospital, Taichung 41354, Taiwan; 4 Department of Physical Therapy, Asia University, Taichung 41354, Taiwan; 5 Department of Pharmacology, School of Medicine, China Medical University, Taichung 40402, Taiwan; 6 Department of Medical Education and Research, China Medical University Beigang Hospital, Yunlin 65152, Taiwan; 7 Department of Neurosurgery, China Medical University Hospital, Taichung 404327, Taiwan; 8 Graduate Institute of Integrated Medicine, China Medical University, Taichung 406040, Taiwan; 9 Department of Neurosurgery, China Medical University Hsinchu Hospital, Hsinchu 302, Taiwan; 10 Department of Medicine, Mackay Medical College, New Taipei 252, Taiwan; 11 College of Pharmacy, Graduate Institute of Natural Products, Kaohsiung Medical University, Kaohsiung 807, Taiwan; 12 Chinese Medicine Research Center, China Medical University, Taichung 406040, Taiwan; 13 Department of Medical Laboratory Science and Biotechnology, College of Health Science, Asia University, Taichung 41354, Taiwan; 14 Department of Medical Research, China Medical University Hsinchu Hospital, Hsinchu 302, Taiwan

**Keywords:** esophageal, cancer, visfatin, VEGF-C, lymphangiogenesis

## Abstract

Lymph node metastasis is a recognized prognostic factor in esophageal cancer. Adipokines, including visfatin, and the molecule vascular endothelial growth factor (VEGF)-C, are implicated in lymphangiogenesis, but whether any association exists between esophageal cancer, adipokines and VEGF-C is unknown. We examined the relevance of adipokines and VEGF-C in esophageal squamous cell carcinoma (ESCC) in the Gene Expression Omnibus (GEO) and The Cancer Genome Atlas (TCGA) databases. We found significantly higher levels of visfatin and VEGF-C expression in esophageal cancer tissue than in normal tissue. Immunohistochemistry (IHC) staining identified that higher levels of visfatin and VEGF-C expression were correlated with advanced stage ESCC. Visfatin treatment of ESCC cell lines upregulated VEGF-C expression and VEGF-C-dependent lymphangiogenesis in lymphatic endothelial cells. Visfatin induced increases in VEGF-C expression by activating the mitogen-activated protein kinase kinases1/2-extracellular signal-regulated kinase (MEK1/2-ERK) and Nuclear Factor Kappa B (NF-κB) signaling cascades. Transfecting ESCC cells with MEK1/2-ERK and NF-κB inhibitors (PD98059, FR180204, PDTC, and TPCK) and siRNAs inhibited visfatin-induced increases in VEGF-C expression. It appears that visfatin and VEGF-C are promising therapeutic targets in the inhibition of lymphangiogenesis in esophageal cancer.

## INTRODUCTION

Esophageal cancer is a highly lethal malignancy that accounted for approximately 5.5% of all cancer deaths worldwide in 2020 [1]. Asian countries have higher incidence and mortality rates of esophageal cancer than other global regions [2]. Taiwan’s 2019 Cancer Registry Annual Report listed esophageal cancer as the ninth most common cause of cancer death nationwide; the leading histologic subtype was squamous cell carcinoma (SCC), accounting for 91.4% of all subtypes [3]. Lymphovascular invasion within the tumor and lymph node metastasis are indicators of poor prognosis [4, 5]. Even in superficial esophageal carcinoma, patients with lymphovascular invasion within the tumor have higher rates of lymph node metastasis (hazard ratio (HR) 5.72) and lower overall survival (HR 1.85) compared with patients without lymphovascular invasion [6]. Spreading of the tumor to adjacent tissues, regional lymph nodes or distant organs are all independent prognostic factors of survival and are included in the cancer staging categories for esophageal cancer of the 8th edition of the American Joint Committee on Cancer (AJCC) [7]. The lymphatic system actively participates in metastatic tumor invasion [8].

Obesity is a major health problem in Taiwan, which experienced steep increases in the rates of obesity and morbid obesity between 2013 and 2016 [9, 10]. Much evidence links obesity with an increase in risk of cancer metastasis, particularly renal, prostate, endometrial, breast, colorectal and esophageal cancers [11, 12]. Adipokines, bioactive substances secreted by adipocytes (fat cells), play important roles in inflammation, metabolic disease, cardiovascular disease, cancer progression and metastasis [13–15]. Adipokines may even induce the epithelial-to-mesenchymal transition (EMT) process in the tumor microenvironment [16]. The relationship between adipokines and lymphangiogenesis has been described in recent studies [17–19]. For example, the adipokine visfatin regulates tumor proliferation, angiogenesis, metastasis and drug resistance in several different types of cancers [20].

Visfatin is regarded as an extracellular nicotinamide phosphoribosyltransferase (eNAMPT) enzyme and a multifunctional adipokine that was first identified in visceral adipose tissue [21]. Upregulated serum levels of visfatin are found in patients with various types of cancers [22, 23]. Visfatin plays a pivotal role in cancer progression and drug resistance [20]. For instance, visfatin appears to lower doxorubicin sensitivity in small cell lung cancer (SCLC) A549 and H1793 cell lines by activating the Akt/ABCC1 signaling pathways [24]. Moreover, levels of visfatin protein and mRNA expression are significantly increased in doxorubicin-resistant non-small cell lung cancer (NSCLC) cell lines [24]. Higher levels of visfatin expression correlate with poorer prognoses in breast, gastric, urothelial, and head and neck SCC [20, 25, 26]. This study examined the role of visfatin in ESCC.

Several molecules have been implicated in lymphangiogenesis [27], including vascular endothelial growth factor (VEGF)-C [28]. High levels of VEGF-C expression correlate with advanced stage disease, deeply invasive tumors and lymph node metastasis [29]. Higher levels of VEGF-C expression are linked to lower 5-year survival rates in esophageal squamous cell carcinoma (ESCC) [30, 31]. Lymphangiogenic mediators are regulated by various signal transduction pathways in cancer, such as the MEK1/2-ERK and NF-κB pathways [32, 33]. In specific, the MEK1/2-ERK and NF-κB signaling cascades is crucial for, cell survival and resistance of chemotherapy and promoting tumor-induced angiogenesis [34]. Thus, examining the MEK1/2-ERK and NF-κB pathways is expected to improve our understanding as to how to reduce lymphangiogenesis cytokine expression in ESCC. This study investigated cellular and molecular mechanisms of visfatin and VEGF-C in ESCC cells. Our findings reveal that visfatin upregulates VEGF-C expression in ESCC cells via the MEK1/2-ERK and NF-κB signaling cascades.

## RESULTS

### Higher levels of visfatin expression in ESCC versus normal tissue

**Figure 1:**
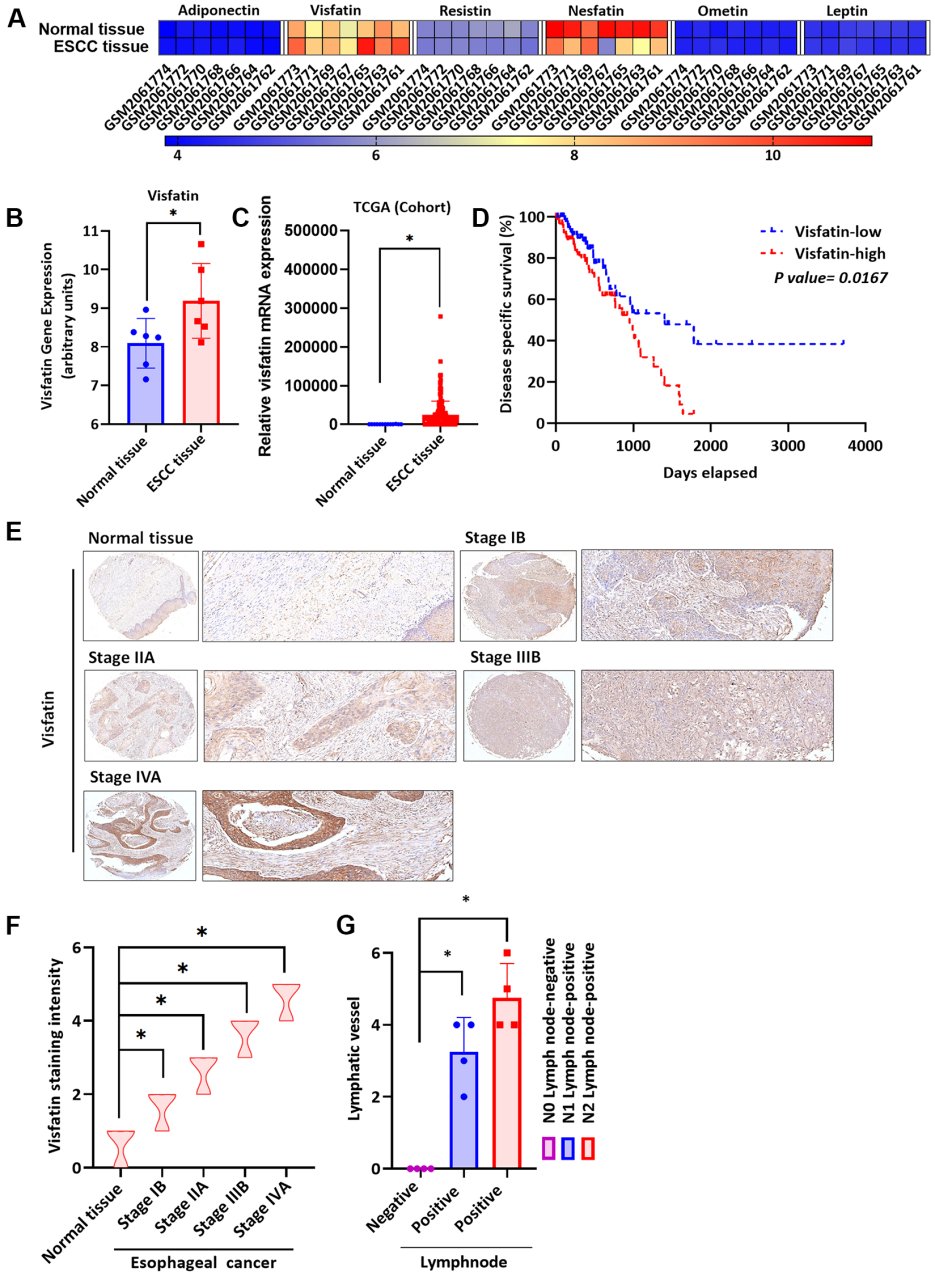
**Clinicopathologic features of visfatin expression in human ESCC tissue samples.** (**A**) The gene expression profiles of visfatin in ESCC tissue and normal tissue samples were analyzed in specimens from the GEO and TCGA databases. (**B**, **C**) Levels of visfatin were significantly increased in ESCC samples compared with normal tissue samples. (**D**) Kaplan-Meier analysis of overall survival according to visfatin expression in patients with esophageal cancer. (**E**, **F**) The human ESCC tissue array specimens were subjected to IHC evaluations with visfatin antibody, and levels of positive staining were quantified by IHC scoring (*N* = 4 per group). Scale bar: 100 μm. (**G**) Positive peritumoral lymphatic vessel density in patients with N0, N1, or N2 ESCC tissue array samples (*N* = 4 per group). ^*^P < 0.05 compared with normal tissue samples or N0 lymph node negative tissue array samples.

Visfatin stimulates the progression of cancers, including breast cancer [35], oral squamous cell carcinoma [36] and gastric cancer [37], but its role in ESCC is unknown. We therefore screened gene expression profiling records from the Gene Expression Omnibus (GEO) database for several adipocytokines, including adiponectin, resistin, nesfatin, omentin and leptin (Figure 1A). Significantly higher levels of visfatin expression were found in ESCC tissues than in normal tissue samples (Figure 1A, 1B). The results were similar in clinical samples downloaded from The Cancer Genome Atlas (TCGA) database (Figure 1C). The 4-year Kaplan-Meier overall survival rates were significantly shorter for the high visfatin expression group compared with the low visfatin expression group (Figure 1D). Tissue array data revealed higher levels of visfatin expression in more advanced ESCC samples than in lower-grade disease samples (Figure 1E). The quantification of these results showed significantly higher levels of visfatin expression in the higher-stage tumors (IIB and IVA) than in the lower-stage tumors (IB and IIA) and normal tissue samples (Figure 1F), indicating a positive association between levels of visfatin expression and ESCC cancer progression. Further experiments identified higher levels of positive peritumoral lymphatic vessel density (LVD) in N1 and N2 stage disease than in N0 tissue samples (Figure 1G and Supplementary Figure 1), indicating that visfatin is associated with lymphatic metastasis in ESCC progression.

### Higher levels of VEGF-C expression in ESCC tissue versus normal tissue

**Figure 2:**
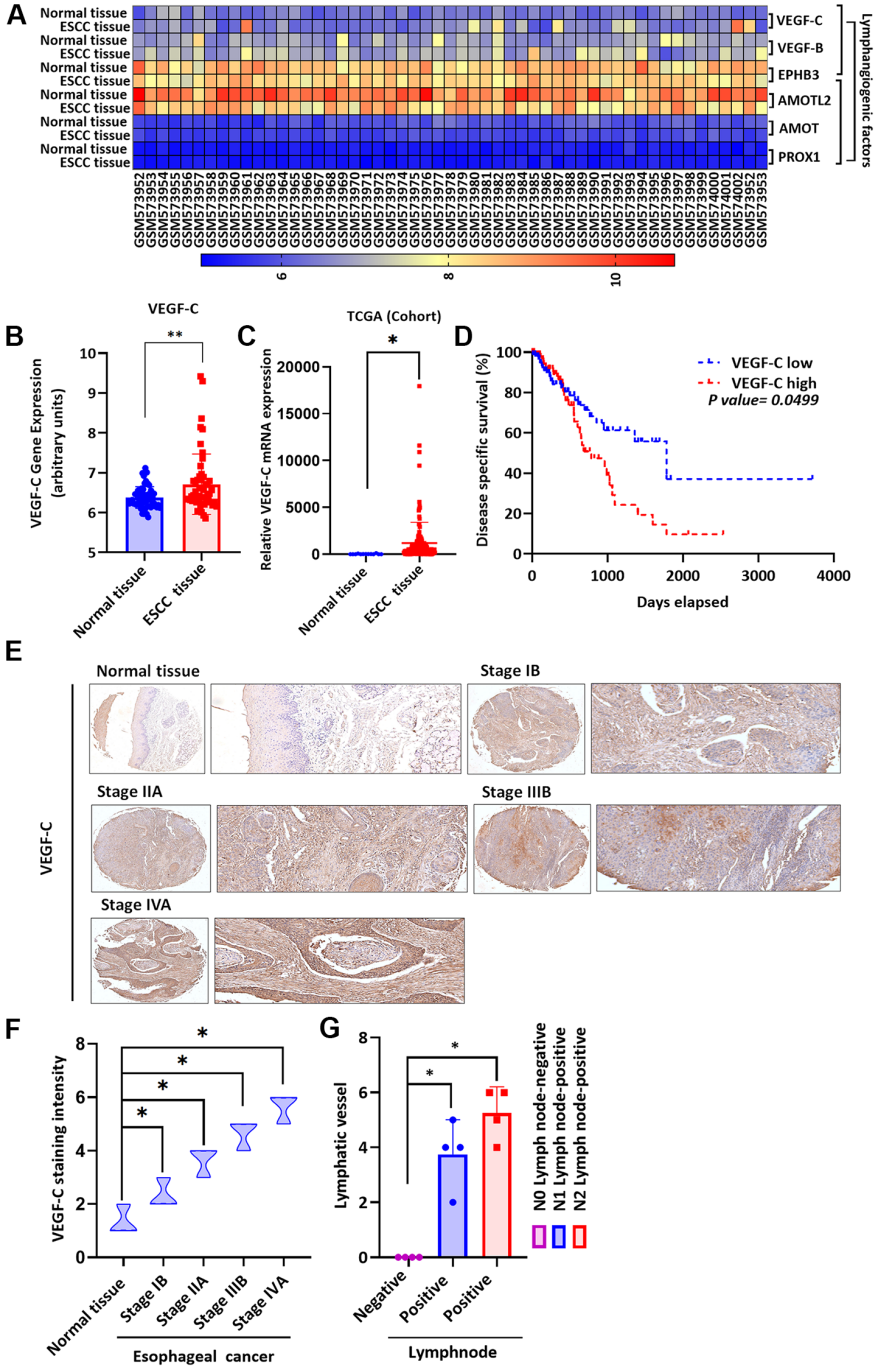
**Clinicopathologic features of VEGF-C expression in human ESCC tissue.** (**A**) The gene expression profiles of VEGF-C in ESCC tissue and normal tissue samples were analyzed in GEO and TCGA database records. (**B**, **C**) Levels of VEGF-C expression were significantly higher in ESCC samples compared with the normal tissue samples. (**D**) Kaplan-Meier analysis of overall survival according to VEGF-C expression in patients with esophageal cancer. (**E**, **F**) The human ESCC tissue array specimens were subjected to IHC evaluations with VEGF-C antibody, and the positive staining was quantified by IHC scoring (*N* = 4 per group). Scale bar: 100 μm. (**G**) Positive peritumoral lymphatic vessel density in patients with N0, N1, or N2 ESCC tissue array samples (*N* = 4 per group). ^*^P < 0.05 compared with normal tissue samples or N0 lymph node negative tissue array samples.

Levels of lymphangiogenic factors, including VEGF-C, are higher in cancers that have metastasized [29]. As VEGF-C is known to regulate lymphangiogenesis in various types of cancer cells [38, 39], we therefore screened gene expression profiling records from the GEO database for several lymphangiogenic genes, including vascular endothelial growth factor-C (*VEGF-C*), *VEGF-B*, ephrin type-B receptor-3 (*EPHB3*), angiomotin-like protein-2 (*AMOTL2*), angiomotin (*AMOT*) and prospero homeobox-1 (*PROX1*) (Figure 2A). Significantly higher levels of VEGF-C expression were found in ESCC tissues than normal tissue samples (Figure 2A, 2B). Similarly, TCGA database screening identified higher levels of VEGF-C expression in ESCC tissues than in normal tissue samples (Figure 2C). Four-year Kaplan-Meier overall survival was shorter in the high VEGF-C expression group compared with the low VEGF-C expression group (Figure 2D). Tissue array results revealed higher levels of VEGF-C expression in patients with higher-grade ESCC than in those with lower-grade disease (Figure 2E); Figure 2F shows significantly higher levels of VEGF-C expression in the higher-stage tumors (IIB and IVA) than in the lower-stage tumors (IB and IIA) and normal tissue samples. Further experiments identified higher levels of positive peritumoral LVD in N1 and N2 stage disease compared with N0 tissue samples (Figure 2G and Supplementary Figure 1), indicating that VEGF-C is associated with lymphatic metastasis in ESCC progression. A positive correlation was observed between levels of visfatin and VEGF-C staining intensity in human ESCC tissue samples (R = 0.9564, Supplementary Figure 2).

### Visfatin induces increases in VEGF-C expression in ESCC

**Figure 3:**
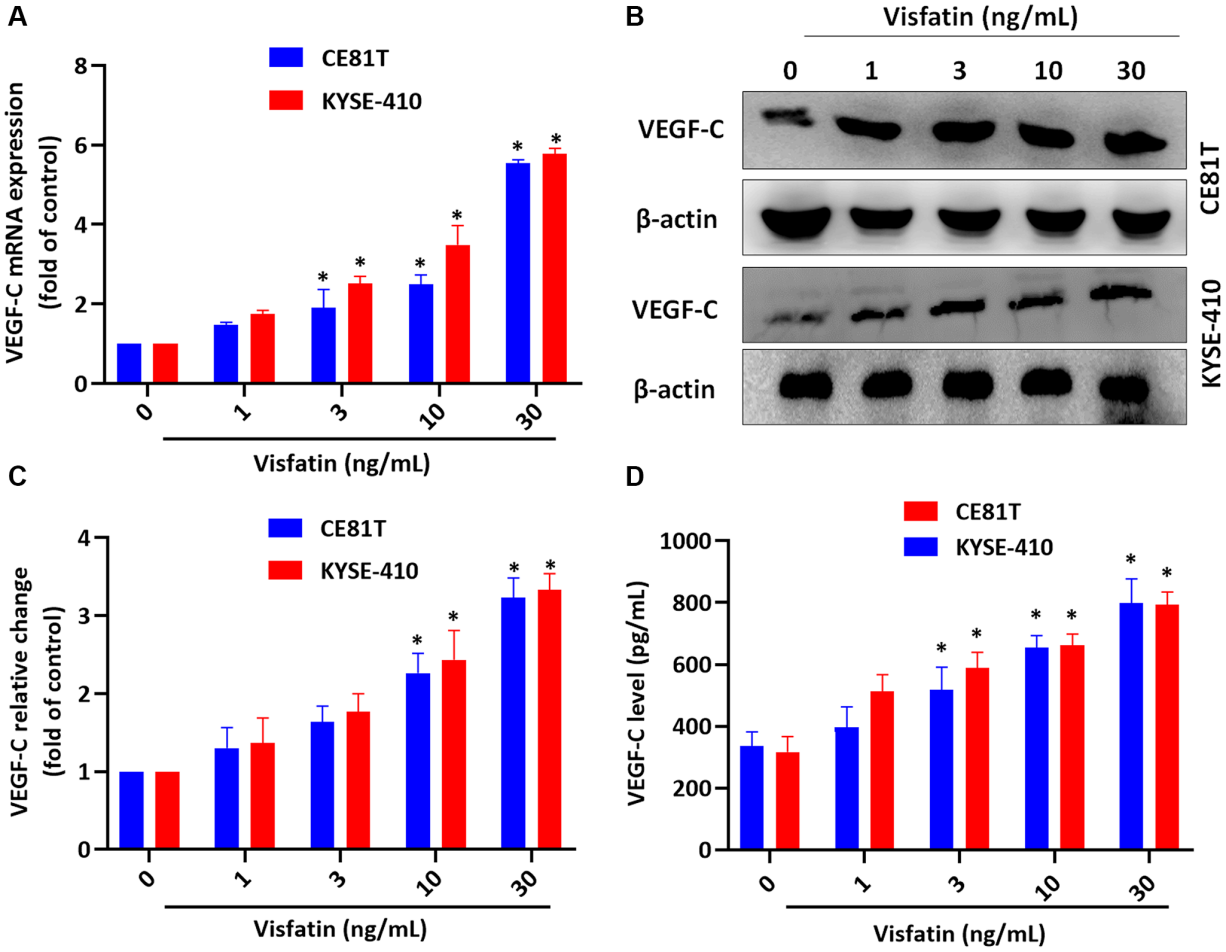
**Visfatin promotes increases in VEGF-C expression in ESCC cells.** (**A**–**D**) ESCC cells were stimulated with visfatin for 24 h, before determining levels of VEGF-C mRNA and protein expression by qPCR (**A**), Western blot (**B**, **C**), and ELISA (**D**). ^*^P < 0.05 compared with the control group.

We first investigated the effects of different visfatin concentrations (1, 3, 10, or 30 ng/mL) upon the viability of the ESCC cell lines CE81T and KYSE-410 (Supplementary Figure 3); only the highest concentration (30 ng/mL) was used in pathway screening analyses and the lymphatic endothelial cell (LEC) tube formation assay. Incubation of the cells with visfatin (1, 3, 10, or 30 ng/mL) significantly increased levels of VEGF-C mRNA and protein expression in ESCC cells (Figure 3A–3D).

### Visfatin induces VEGF-C-dependent lymphangiogenesis

**Figure 4:**
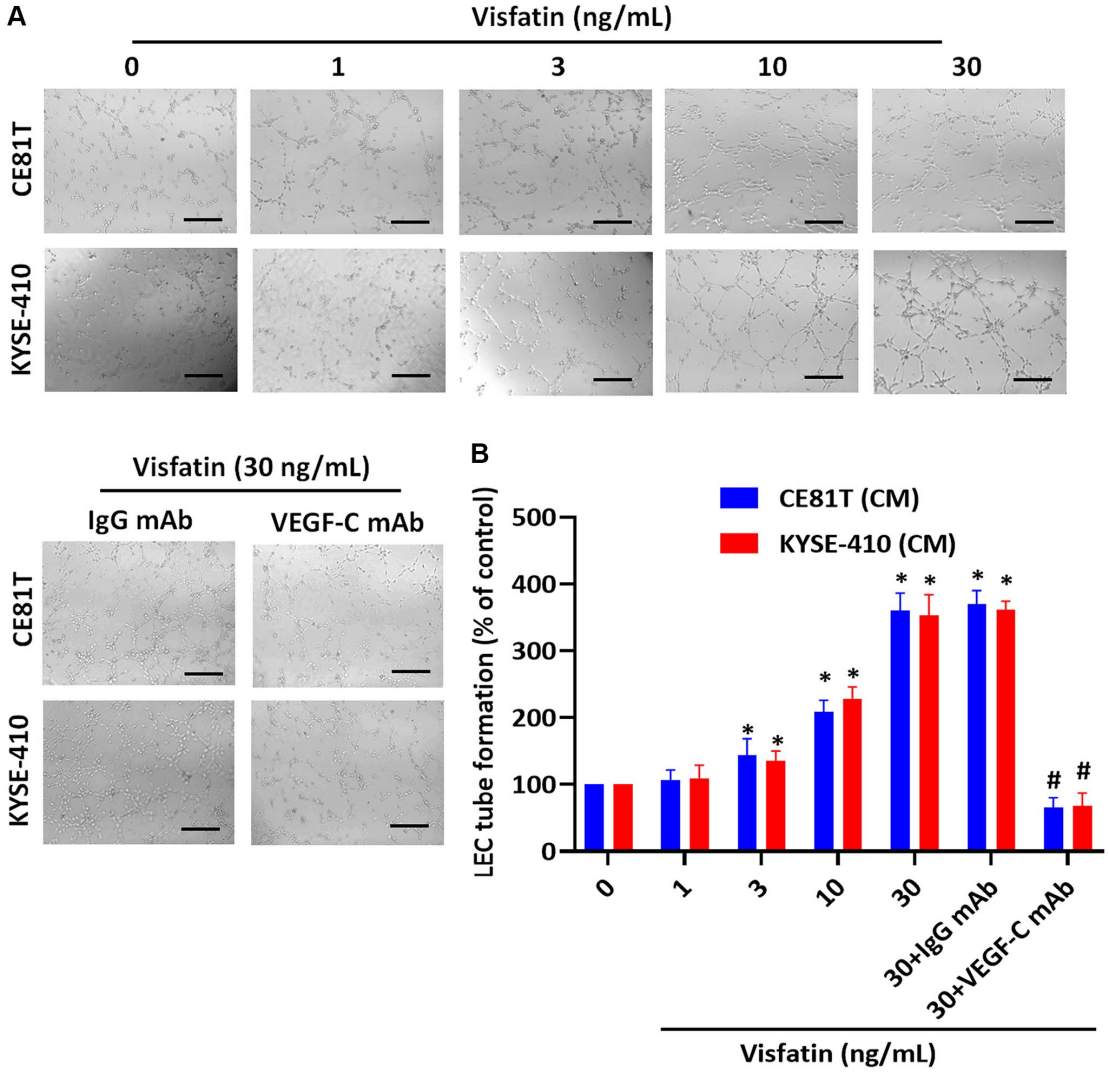
**Visfatin stimulates VEGF-C-dependent lymphangiogenesis in ESCC cells.** (**A**, **B**) ESCC cells were stimulated with visfatin for 24 h, or preincubated with IgG control antibody or VEGF-C antibody (1 μg/mL) for 30 min, then incubated with visfatin (30 ng/mL) for 24 h. CM was collected from each experiment and added to LECs, to examine tube formation activity. ^*^P < 0.05 compared with the control group; ^#^*P* < 0.05 compared with the visfatin-treated group.

We then examined whether visfatin plays a role in VEGF-C-regulated lymphangiogenesis. Conditioned medium (CM) from ESCC cells promoted tube formation activity in LECs (Figure 4A). VEGF-C monoclonal antibody (mAb), but not the immunoglobulin (Ig)G control, abolished visfatin-mediated effects (Figure 4A, 4B).

### Visfatin promotes higher levels of VEGF-C expression by activating MEK1/2 signaling

**Figure 5:**
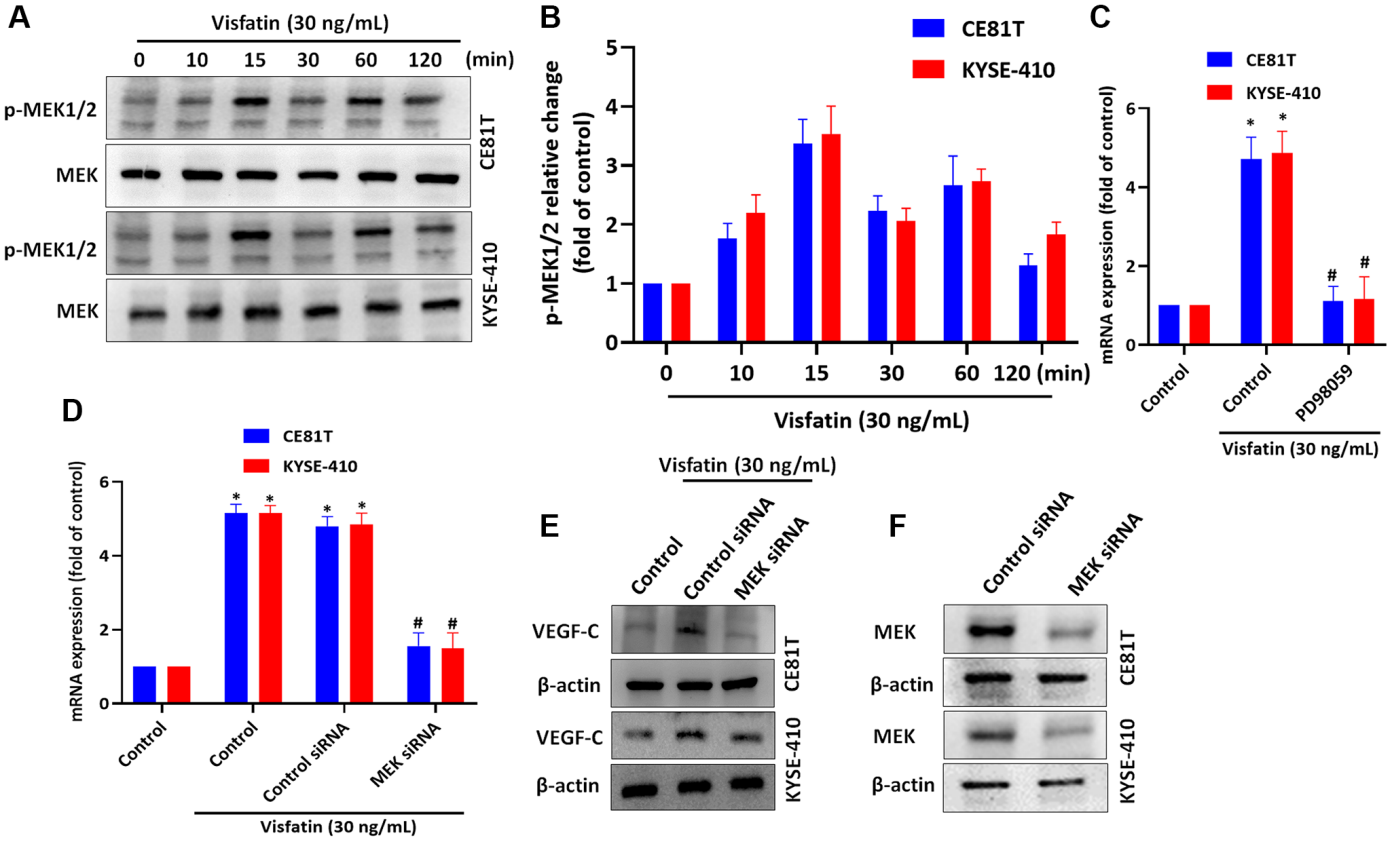
**Visfatin induced increases in VEGF-C expression and lymphangiogenesis by activating MEK1/2 signaling.** (**A**, **B**) ESCC cells were treated with visfatin (30 ng/mL) for the indicated times and then MEK1/2 phosphorylation was examined by Western blot and quantified by ImageJ software. (**C**, **D**) ESCC cells were transfected or preincubated with the MEK1/2 inhibitor PD98059 or siRNAs for 24 h, before determining levels of VEGF-C expression by qPCR. (**E**) ESCC cells were transfected with MEK siRNA for 24 h, then stimulated with visfatin (30 ng/mL) for 24 h. Levels of VEGF-C expression were examined by Western blot. (**F**) ESCCs were transfected with a MEK siRNA and MEK expression was examined by Western blot. *P < 0.05 compared with the control group; ^#^*P* < 0.05 compared with the visfatin-treated group.

MEK signaling is implicated in lymphangiogenesis and metastasis [40, 41]. We therefore incubated the ESCC cell lines with visfatin (30 ng/mL), to examine whether MEK signaling is involved in ESCC lymphangiogenesis. After 15 min of incubation, MEK1/2 phosphorylation levels were increased (Figure 5A, 5B). Pretreating cells with a MEK inhibitor or small interfering RNA (siRNA) blocked visfatin-mediated increases in VEGF-C mRNA and protein expression (Figure 5C–5E). Similar results were observed when ESCCs were transfected with MEK siRNA (Figure 5F).

### Visfatin increases levels of VEGF-C-expression by activating ERK signaling

**Figure 6:**
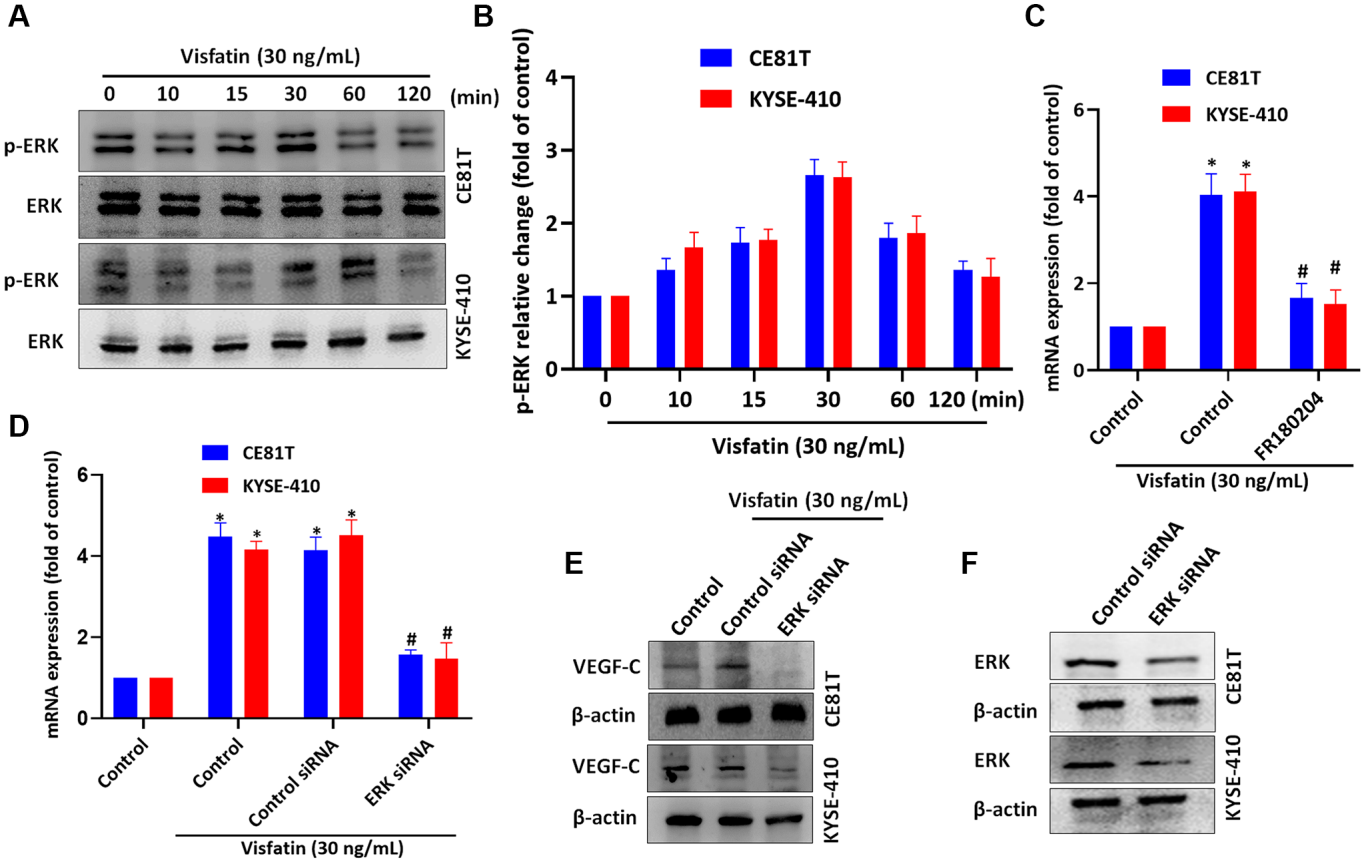
**Visfatin induced increases in levels of VEGF-C expression and lymphangiogenesis by activating ERK signaling.** (**A**, **B**) ESCC cells were treated with visfatin (30 ng/mL) for the indicated times and then ERK phosphorylation was examined by Western blot and quantified by ImageJ software. (**C**, **D**) ESCC cells were transfected or preincubated with the ERK inhibitor FR180204 or siRNAs for 24 h and then VEGF-C expression levels were measured by qPCR. (**E**) ESCC cells were transfected with ERK siRNA for 24 h, then stimulated with visfatin (30 ng/mL) for 24 h. Levels of VEGF-C expression were examined by Western blot. (**F**) ESCCs were transfected with a ERK siRNA and ERK expression was examined by Western blot. ^*^P < 0.05 compared with the control group; ^#^*P* < 0.05 compared with the visfatin-treated group.

We then incubated the ESCC cell lines with visfatin (30 ng/mL), to examine whether ERK signaling is involved in ESCC lymphangiogenesis. ERK phosphorylation levels were significantly increased from baseline in both cell lines after 15 and 30 min (Figure 6A, 6B). Pretreating cells with the ERK inhibitor or siRNA blocked visfatin-mediated increases in VEGF-C mRNA and protein expression (Figure 6C–6E). Similar results were observed when ESCCs were transfected with ERK siRNA (Figure 6F).

### Visfatin induces VEGF-C expression by activating NF-κB signaling

**Figure 7:**
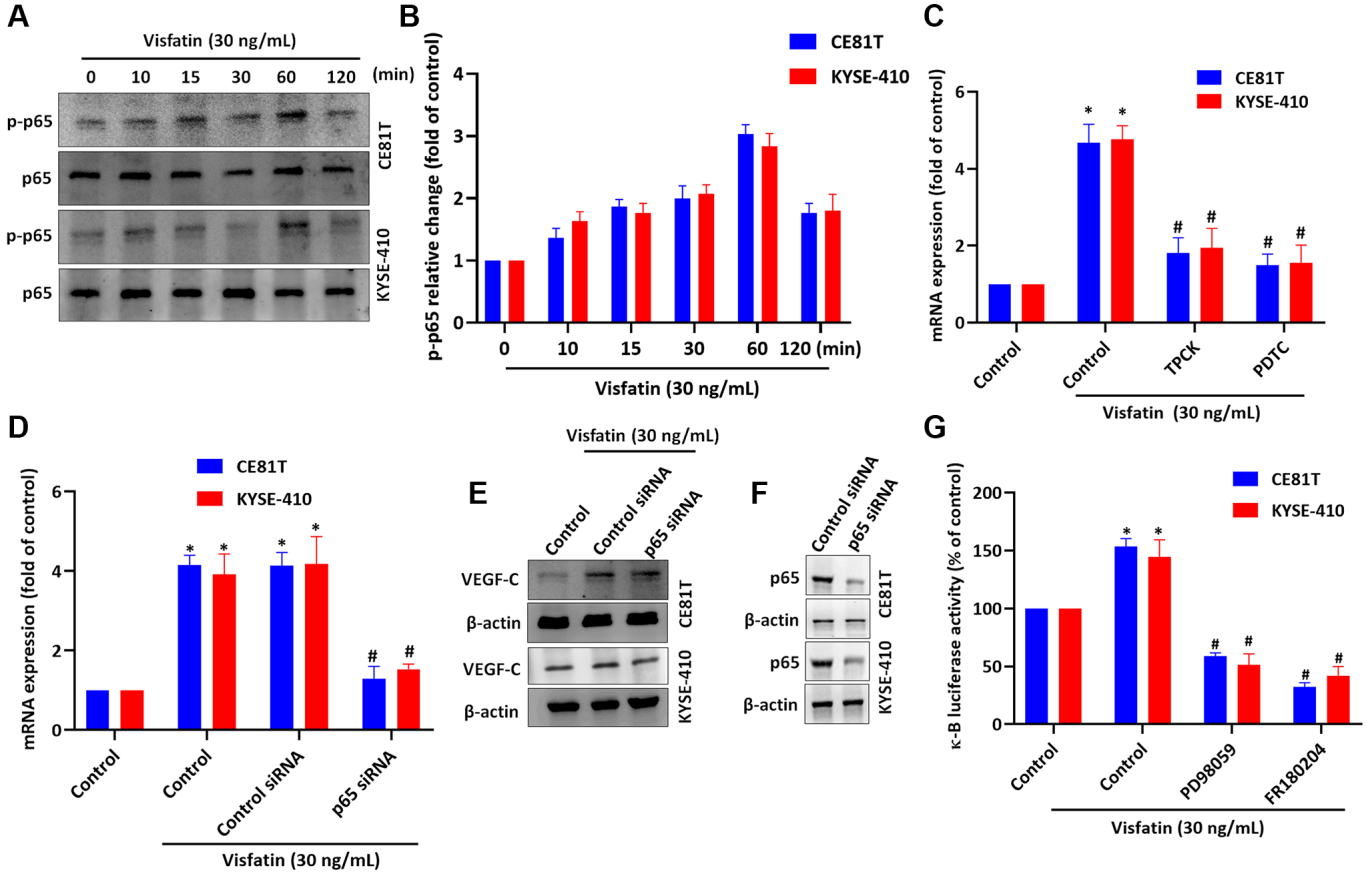
**Visfatin induced increases in VEGF-C expression and lymphangiogenesis by activating NF-κB signaling.** (**A**, **B**) ESCC cells were treated with visfatin (30 ng/mL) for the indicated times and then p65 phosphorylation was examined by Western blot and quantified by ImageJ software. (**C**, **D**) ESCC cells were transfected or preincubated with NF-κB inhibitors (PDTC and TPCK) or siRNAs for 24 h and then VEGF-C expression levels were measured by qPCR. (**E**) ESCC cells were transfected with p65 siRNA for 24 h, then stimulated with visfatin (30 ng/mL) for 24 h. Levels of VEGF-C expression were examined by Western blot. (**F**) ESCCs were transfected with a p65 siRNA and p65 expression was examined by Western blot. (**G**) ESCCs were treated with MEK and ERK inhibitors then stimulated with visfatin, and NF-κB luciferase activity was examined. ^*^P < 0.05 compared with the control group; ^#^*P* < 0.05 compared with the visfatin-treated group.

NF-κB is a well-known transcription factor in cancer progression and metastasis [42] and NF-κB activation regulates ESCC angiogenesis [43]. We therefore incubated the ESCC cell lines with visfatin (30 ng/mL), to examine whether NF-κB signaling is involved in ESCC lymphangiogenesis. The p65 phosphorylation levels were significantly increased from baseline in both cell lines after 60 min (Figure 7A, 7B). Pretreating cells with NF-κB inhibitors or siRNAs blocked visfatin-mediated increases in VEGF-C mRNA and protein expression (Figure 7C–7E). Similar results were observed when ESCCs were transfected with p65 siRNA (Figure 7F). In addition, stimulation of ESCCs with visfatin enhanced NF-κB luciferase activity, which was reversed by MEK and ERK inhibitors (Figure 7G). Thus, visfatin appears to upregulate VEGF-C by activating the MEK, ERK and NF-κB signaling cascades.

## DISCUSSION

Esophageal cancer is a relatively common cancer worldwide and is well recognized for its metastatic potential and poor prognosis [44]. Around 90% of esophageal cancers in Asia are the ESCC subtype, which has a particularly poor prognosis and high mortality rate [2, 45]. The 5-year survival rate for people with esophageal cancer is quite low (~25%), despite significant advancements in diagnosis and therapy [46]. Improved treatment strategies and targets may help to reduce the high mortality rate in esophageal cancer [47].

Adipocytokines are implicated in the carcinogenesis, progression, recurrence, and metastasis of different cancers [48]. Patients with ESCC and EA presented lower adiponectin levels than controls. In brief, patients with EA had significantly lower adiponectin than those with ESCC [49], while lower resistin mRNA expression was identified in ESCC tissue and serum compared with normal esophageal tissues [50]. In addition, levels of serum nesfatin-1 were lowered in lung cancer patients than in healthy subjects [50], the same case were found in ometin-1 [51]. Moreover, the levels of leptin were significantly correlated with lymph node involvement and advanced tumor stage esophageal squamous cell carcinoma [52]. Visfatin in particular appears to have a vital role in cancer and inflammation [53, 54]. The visfatin-neurogenic locus notch homolog protein 1 (Notch-1) pathway contributes to breast cancer development by activating NF-κB signaling [55], while high circulating visfatin levels reportedly significantly increase the risk of cancer [56]. Visfatin also appears to promote chondrosarcoma metastasis [57]. In addition, plasma visfatin levels are elevated in patients with type 2 diabetes mellitus [58], while serum visfatin levels are elevated in the peripheral blood of patients with breast cancer [59]. In this study, we found increasingly higher levels of visfatin corresponding with higher disease stage in ESCC tissue compared with normal tissue. Thus, visfatin may serve as a new therapeutic target in the treatment of cancer metastasis.

Lymphangiogenesis favors the development of cancer metastasis [60]. Increased levels of lymphangiogenic genes promote tumor relapse and poor prognosis, and thus serve as potential targets for preventing lymphatic metastasis [61]. This is supported by our study evidence, which identified that lymphangiogenic gene expression in ESCC clinical samples has clinical significance for survival. Other research has also reported that visfatin stimulates the production of human endothelial VEGF and matrix metalloproteinases (MMP-2 and MMP-9) in human umbilical vein endothelial cells [62]. In our study, visfatin significantly and dose-dependently upregulated levels of VEGF-C gene expression and protein production in ESCC cells. Visfatin also dose-dependently promoted LEC tube formation. Interestingly, visfatin-mediated lymphangiogenesis was significantly inhibited by VEGF-C mAb treatment. Our findings offer novel insights into the effects of visfatin upon VEGF-C-dependent lymphangiogenesis in ESCC.

The mitogen-activated protein kinase (MAPK) signaling pathways play crucial roles in the survival of disseminated tumor cells and cancer drug resistance [63]. Several drugs have been developed that specifically target the MAPK signaling pathway network; these drugs help to overcome cancer cell drug resistance and sensitivity [41]. Previous studies have demonstrated that MAPK signaling pathways promote VEGF-A secretion and angiogenesis, as well as osteosarcoma metastasis [64]. Notably, MAPK signaling regulates angiogenic and lymphangiogenic cytokine production in head and neck SCC [32]. In this study, MEK1/2-ERK inhibitors and siRNAs reversed visfatin-induced stimulation of VEGF-C expression.

NF-κB is a critical transcription factor in cancer [65] and cancer-associated disease [66]. Targeting NF-κB activity is a prominent strategy in the treatment of various cancers [67], including esophageal adenocarcinoma [68]. NF-κB activation is also associated with chemoresistance and the metastasis of esophageal cancer [69, 70]. The NF-κB signaling pathway is constitutively activated in ESCC cell lines and RNA interference targeting at p65 increases the sensitivity of ESCC cell lines to 5-fluorouracil chemotherapy [71]. In addition, higher levels of NF-κB protein expression in esophageal cancer tissue compared with adjacent normal esophageal mucosa [72]. Our findings indicate the involvement of p65 phosphorylation in visfatin-induced production of VEGF-C. According to our evidence, visfatin-mediated production of VEGF-C is critical in ESCC progression and MEK1/2-ERK and NF-κB signaling is critical for ESCC lymphangiogenesis. No specific receptor has yet been identified for visfatin, although visfatin activity appears to be mediated by enzymatic activity [73–75] and also by an insulin receptor [76].

Finally, it should be noted that several limitations exist in this study. Firstly, although our data strongly suggest that visfatin enhances VEGF-C-dependent LEC tube formation in ESCCs, we cannot exclude the possibility that crosstalk exists between visfatin and its receptor, so we would like to address this limitation in future projects. Secondly, the impact of NF-κB on ESCC progression and lymphangiogenesis needs to be assessed in animal disease models. We hope to use NF-κB inhibitors to study *in vivo* ESCC progression and lymphangiogenesis. Thirdly, our data strongly suggest that visfatin and VEGF-C expression levels were higher in lymph node positive than in lymph node negative ESCC tissues, we cannot exclude the possibility that address the accurate observation of lymphatic vessel infiltration. Primarily, access to tumor tissue may not always be feasible due to increasingly competing demands for tumor tissue in research and clinical practice.

## CONCLUSIONS

**Figure 8:**
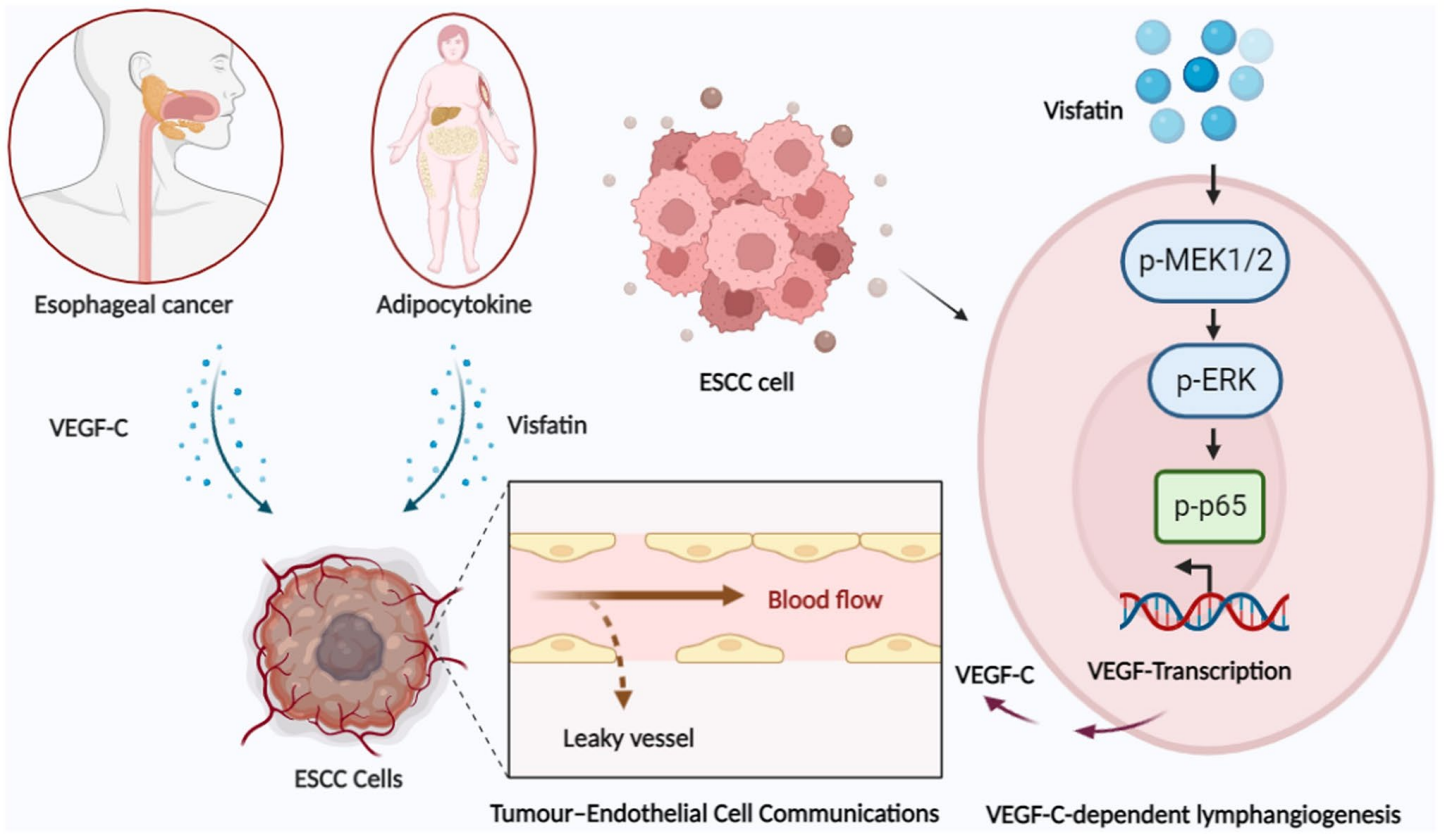
Schematic illustration of how signaling pathways participate in visfatin-induced stimulation of VEGF-C expression and the subsequent stimulation of ESCC lymphangiogenesis in esophageal cancer.

In this study, data from the GEO and TCGA databases demonstrated significantly higher levels of visfatin and VEGF-C expression in ESCC tissue samples compared with levels in adjacent normal tissue, and positive correlations were observed between visfatin and VEGF-C expression with ESCC clinical disease stages. Our experiments indicate that the MEK1/2-ERK and NF-κB pathways are involved in visfatin-mediated upregulation of VEGF-C and VEGF-C-dependent lymphangiogenesis in ESCC cells (Figure 8). Thus, visfatin and VEGF-C may represent new molecular therapeutic targets for inhibiting ESCC lymphangiogenesis.

## MATERIALS AND METHODS

### Materials

Recombinant human visfatin was purchased from PeproTech, Inc. (Rocky Hill, NJ, USA). VEGF-C (sc-9047) and IgG (sc-69786) monoclonal antibodies were purchased from Santa Cruz Biotechnology (Santa Cruz, CA, USA). The cell culture mediums Dulbecco’s Modified Eagle Medium (DMEM) and Roswell Park Memorial Institute (RPMI) 1640 were purchased from Gibco Life Technologies Corporation (Grand Island, NY, USA). Chloroform and isopropanol were purchased from J.T. Baker (NJ, USA). Oligo-dT was obtained from MDBio Inc. (Gaithersburg, Maryland, USA). Dithiothreitol (DTT), dNTP, MMLV and 5X first-strand buffer were purchased from Invitrogen Corporation (Carlsbad, California, USA). Taqman^®^ One-Step PCR Master Mix, qPCR primers and probes were bought from Applied Biosystems (Foster City, CA, USA). A BCA protein assay kit was obtained from Pierce (Meridian Rd. Rockford, IL 61101 USA). Tri’s buffer, 30% acrylamide and sodium dodecyl sulfate-polyacrylamide gel electrophoresis **(**SDS-PAGE) were purchased from Amresco Inc (6681 Cochran Rd, Solon, Ohio, USA). The detailed source of inhibitors and siRNAs are listed in Supplementary Tables 1 and 2. All other chemical reagents not already mentioned were obtained from Sigma-Aldrich (St. Louis, MO, USA).

### Cell culture

The well-differentiated human ESCC cell line CE81T/VGH was obtained from the Bioresource Collection and Research Center (BCRC, Hsinchu, Taiwan, ROC) and cultured in DMEM. The poorly differentiated invasive ESCC cell line KYSE-410 was obtained from the European Collection of Cell Culture (ECACC^®^) **(**Porton Down, SP4 0JG Salisbury, UK) and cultured in RPMI. The culture medium contained 10% fetal bovine serum (FBS) (Lonza, Walkersville, MD, USA) and streptomycin/penicillin (PS) 100 U/mL.

Human telomerase gene-immortalized human dermal lymphatic endothelial cells (hTERT-HDLECs), an immortalized human LEC line, was purchased from Lonza (Walkersville, MD, USA). The LECs were grown in EGM-2 MV BulletKit Medium, consisting of endothelial basal medium-2 (EBM-2) plus a SingleQuots kit (Lonza). Cells were seeded onto culture dishes precoated with 1% gelatin. Cell incubation was conducted in a humidified atmosphere of 37°C, 5% CO_2_ [39, 77].

### Bioinformatics analysis

The GSE23400 dataset was downloaded from the GEO database and examined for levels of visfatin and VEGF-C expression in human ESCC and normal tissue samples. Differential gene expression analysis from the TCGA database has identified an inverse relationship between visfatin and VEGF-C transcript levels in ESCC and adjacent normal tissue samples [78, 79].

### Cell viability assay

ESCC cells were incubated with indicating concentrations of visfatin for 24 h then cell viability was determined with the 3-(4,5-dimethylthiazol-2-yl)- 2,5-diphenyltetrazolium bromide (MTT) assay over 2 h. Dimethyl sulfoxide (DMSO) was applied, and cell viability were detected on a microplate reader.

### Quantitative real-time PCR

Total RNA was extracted from ESCC cells using TRIzol™ Reagent (MDBio, Taipei, Taiwan) and RNA quality was analyzed by a NanoVue Plus™ Spectrophotometer (Biochrom Ltd., Cambridge, UK). A MMLV Kit (Thermo Fisher Scientific; Waltham, MA, USA) used 1-3 (μg/μL) of total RNA to convert RNA to cDNA. The converted cDNA was amplified with primers (primers used in the qPCR assays are listed in Supplementary Table 3) using the StepOnePlus™ Real-Time PCR System (Applied Biosystems, Foster City, CA, USA) [57, 80, 81].

### Western blot

Total proteins were extracted using RIPA lysis buffer containing protease inhibitors, then quantified with the BCA Protein Assay Kit (Thermo Fisher Scientific Inc., Waltham, MA, USA). 30 μg of total proteins were separated by SDS-PAGE electrophoresis then transferred to polyvinylidene difluoride (PVDF) membranes (Millipore, Bedford, MA, USA). The blots were blocked with 4% BSA, then incubated with primary antibodies overnight (antibodies used in the Western blot assays are listed in Supplementary Table 4). The blots were then incubated with horseradish peroxidase (HRP) conjugated secondary antibodies at room temperature for 1 h. Enhanced chemiluminescent imaging of the blots was visualized by the UVP Biospectrum system (UVP, Upland, CA, USA) [82–84].

### ELISA

ESCC cells were plated in 6-well dishes and grown to confluence. The culture medium was then exchanged with serum-free RPMI or DMEM medium. Cells were treated for 24 h with visfatin (0, 1, 3, 10, or 30 ng/mL). CM was collected and the levels of VEGF-C expression were examined using a human VEGF-C ELISA kit (R&D Systems, MN, USA) according to the manufacturer’s protocol [79, 85].

### Tube formation

Matrigel (BD Biosciences; Bedford, MA, USA) was dissolved at 4°C, then added at a concentration of 100 μL to each well of 48-well plates and incubated at 37°C for 30 min. Briefly, LECs were resuspended in MV2 serum-free medium and mixed with the CM from the visfatin-treated cells (0, 1, 3, 10, or 30 ng/mL), before being added to the wells. After 6–8 h of incubation at 37°C, LEC tube formation was examined by microscopy. Tube branches and lengths were examined at a magnification of 20X and quantified by MacBiophotonics ImageJ software (v1.51, National Institutes of Health, Bethesda, MD, USA) [79, 86].

### Tissue array

A Human ESCC Tissue Array was supplied by US Biomax (Derwood, Maryland, USA). The sections were deparaffinized with xylene and rehydrated with ethanol for immunohistochemistry (IHC) staining [87–89]. The sections were immunoassayed with visfatin and VEGF-C antibodies (1:200) overnight, then incubated with secondary antibody (1:200) for 1 h at room temperature. Finally, all tissues were stained with 3,3-diaminobenzidine and photographed using a ImageXpress Pico (Molecular Devices, San Jose, CA, USA). Assessment of lymphatic vessels in N0, N1 and N2 tumor tissue were reviewed independently in a blinded manner by two observers. Assessments at different parameters of the tumor tissue sought to identify vascular hotspots and positive microvessels. Counting was repeated for the whole set of tissue sections to quantify the reproducibility of the methodology. Levels of visfatin and VEGF-C expression are described using a scoring system including staining intensity and percentages of stained tumor cells. Moderate or strong staining was assessed as a positive expression of visfatin or VEGF-C in tumor tissues.

### NF-κB luciferase assay

The NF-κB luciferase plasmid (Stratagene; St. Louis, MO, USA) was transfected into ESCCs using Lipofectamine™ 2000 Transfection Reagent (Thermo Fisher Scientific, Carlsbad, CA, USA), followed by treatment with pharmacological inhibitors of MEK and ERK. The Dual-luciferase^®^ Reporter Assay System was used to examine luciferase activity (Promega, Madison, WI, USA).

### Statistical analysis

All statistical analyses were performed using GraphPad Prism version 5.0 (GraphPad Software). All results are expressed as the mean ± standard deviation (SD) of at least three independent experiments. The Student’s *t*-test compared the means between experimental groups. The statistical difference was significant if the *p*-value was < 0.05.

## Supplementary Materials

Supplementary Figures

Supplementary Tables
