# Supplementary material for: Visfatin upregulates VEGF-C expression and lymphangiogenesis in esophageal cancer by activating MEK1/2-ERK and NF-κB signaling
Source: Aging (Albany NY). 2023 Jun 7;15(11):4774–93. doi: 10.18632/aging.204762 (PMC10292883; doi:10.18632/aging.204762)
Supplement: Supplementary Figures [file aging-15-204762-s001.pdf]

SUPPLEMENTARY FIGURES

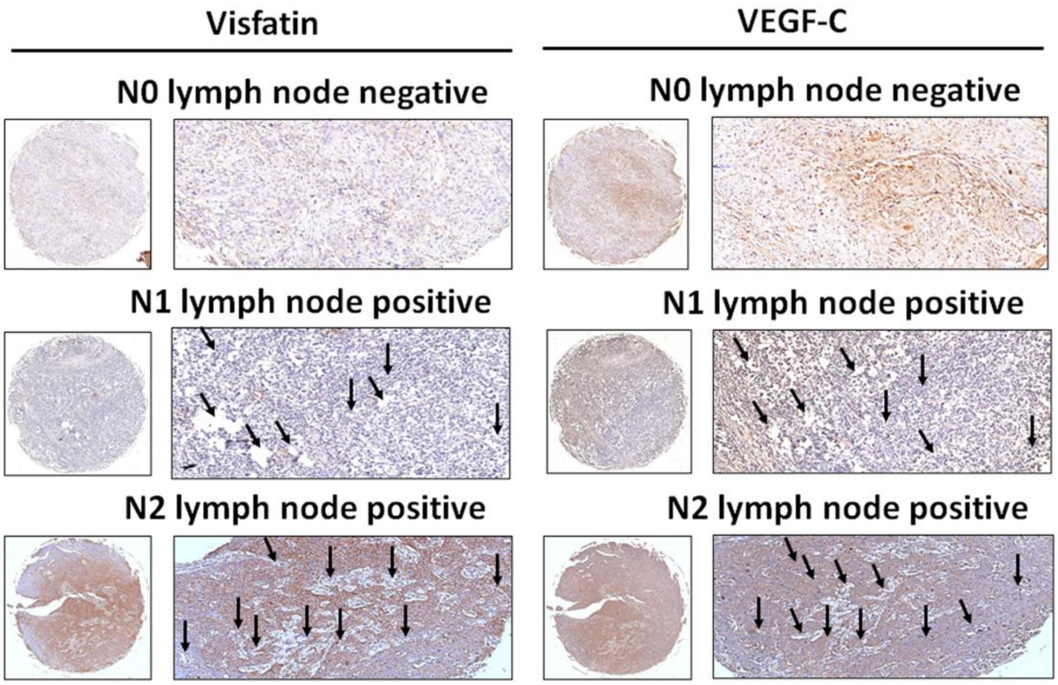

Supplementary Figure 1. Lymphatic vessel density levels are upregulated in ESCC tumor tissues. Histologic sections of ESCC tumor stained with immunoassayed with visfatin or VEGF-C antibody. The black arrows indicate positive peritumoral lymphatic vessels identified in the ESCC tumor tissues.

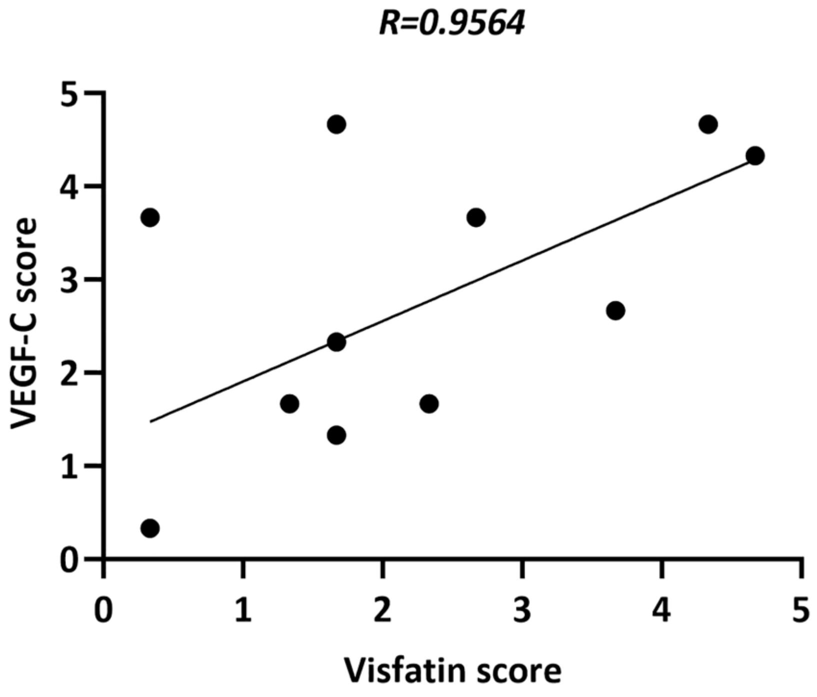

Supplementary Figure 2. A positive correlation was identified between levels of visfatin and VEGF-C expression on ESCC tumor tissues. Histologic sections of ESCC tumor stained with visfatin or VEGF-C antibody and positive association between visfatin and VEGF-C was analyzed.

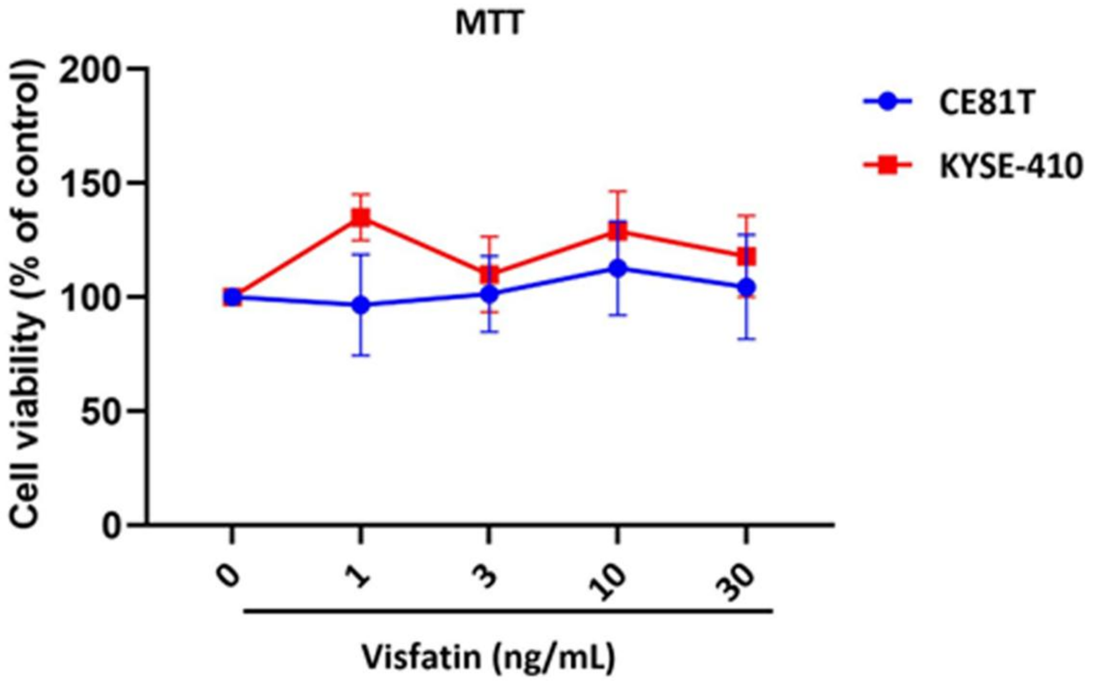

Supplementary Figure 3. Visfatin K does not affect ESCC cell viability. ESCCs were incubated with different concentrations of visfatin K (0, 1, 3, 10, or 30 ng/mL) for 24 h and cell viability was examined by the MTT assay.
