# Supplementary material for: Visfatin upregulates VEGF-C expression and lymphangiogenesis in esophageal cancer by activating MEK1/2-ERK and NF-κB signaling
Source: Aging (Albany NY). 2023 Jun 7;15(11):4774–93. doi: 10.18632/aging.204762 (PMC10292883; doi:10.18632/aging.204762)
Supplement: Supplementary Tables [file aging-15-204762-s002.pdf]

SUPPLEMENTARY TABLES

Supplementary Table 1. Inhibitors used in this study.

Inhibitors	Name	Source	Catalog No.	Concentration
**MEK**	PD98059	Sigma (St. Louis, MO, USA)	P215-1MG	10 μM
**ERK**	FR180204	Santa Cruz Biotechnology, Dallas, TX, USA	SC-203945	10 μM
**κB**	PDTC	Sigma (St. Louis, MO, USA)	P8765-1G	3 μM
**κB**	TPCK	Sigma (St. Louis, MO, USA)	T4376-100MG	3 μM

Supplementary Table 2. siRNA used in this study.

Gene	Species	Source	Catalog no
**MEK**	Human	Dharmacon (2650 Crescent Dr, Lafayette, USA)	L00357100
**ERK**	Human	Dharmacon (2650 Crescent Dr, Lafayette, USA)	L00355500
**p65**	Human	Dharmacon (2650 Crescent Dr, Lafayette, USA)	L-003533-00-0005
**Control**	Human	Dharmacon (2650 Crescent Dr, Lafayette, USA)	D-001810-10-05

Supplementary Table 3. Primers used in this study.

Gene	Forward	Reverse
VEGF-C	CACTTGCTGGGCTTCTTCT	CACAGACCGTAACTGCTCCT
GAPD-H	AATGGACAACTGGTCGTGGA	CCCTCCAGGGATCTGTTTG

Supplementary Table 4. Antibodies used in this study.

Protein	Dilution	Catalog No.	Source
**VEGF-C**	1:3000	SC-9047	Santa Cruz Biotechnology, Dallas, TX, USA
**Visfatin**	1:3000	ab45890	Abcam, Shanghai, China
**β-actin**	1:3000	SC-58673	Santa Cruz Biotechnology, Dallas, TX, USA
**p-MEK1/2**	1:3000	#9121	Cell Signaling (Danvers, MA, USA)
**MEK**	1:3000	#9122	Cell Signaling (Danvers, MA, USA)
**p-ERK**	1:3000	SC-7383	Santa Cruz Biotechnology, Dallas, TX, USA
**ERK**	1:3000	SC-1647	Santa Cruz Biotechnology, Dallas, TX, USA
**p-p65**	1:3000	SC-101752	Santa Cruz Biotechnology, Dallas, TX, USA
**p65**	1:3000	SC-8008	Santa Cruz Biotechnology, Dallas, TX, USA
